# Profibrotic Molecules Are Reduced in CRISPR-Edited Emery–Dreifuss Muscular Dystrophy Fibroblasts

**DOI:** 10.3390/cells14171321

**Published:** 2025-08-27

**Authors:** Eleonora Cattin, Elisa Schena, Elisabetta Mattioli, Stefania Marcuzzo, Silvia Bonanno, Paola Cavalcante, Federico Corradi, Daniela Benati, Giorgia Farinazzo, Marco Cattaneo, Veronica De Sanctis, Roberto Bertorelli, Lorenzo Maggi, Melania Giannotta, Antonella Pini, Gaetano Vattemi, Denise Cassandrini, Marco Cavallo, Cristina Manferdini, Gina Lisignoli, Beatrice Fontana, Ilaria Pace, Claudio Bruno, Roberta Roncarati, Chiara Fiorillo, Manuela Ferracin, Eric C. Schirmer, Alessandra Recchia, Giovanna Lattanzi

**Affiliations:** 1 Department of Life Sciences, University of Modena and Reggio Emilia, 41121 Modena, Italy; eleonora.cattin@unimore.it (E.C.); federico.corradi@unimore.it (F.C.); daniela.benati@unimore.it (D.B.); 2 Centre for Regenerative Medicine, “Stefano Ferrari” University of Modena and Reggio Emilia, 41121 Modena, Italy; 3 CNR Institute of Molecular Genetics “Luigi Luca Cavalli-Sforza”, Unit of Bologna, 40136 Bologna, Italy; elisa.schena@cnr.it (E.S.); e.mattioli@area.bo.cnr.it (E.M.); roberta.roncarati@cnr.it (R.R.); 4 IRCCS Istituto Ortopedico Rizzoli, 40136 Bologna, Italy; 5 Neurology 4—Neuroimmunology and Neuromuscular Diseases, Fondazione IRCCS Istituto Neurologico Carlo Besta, 20133 Milan, Italy; stefania.marcuzzo@istituto-besta.it (S.M.); silvia.bonanno@istituto-besta.it (S.B.); paola.cavalcante@istituto-besta.it (P.C.); giorgia.farinazzo@istituto-besta.it (G.F.); lorenzo.maggi@istituto-besta.it (L.M.); 6 Neurology III—Neuroalgology Unit, IRCCS “C. Besta” Neurologic Institute, 20133 Milan, Italy; marco.cattaneo@istituto-besta.it; 7 PhD Program in Pharmacological Biomolecular Sciences, Experimental and Clinical, University of Milan, 20133 Milan, Italy; 8 Department of Cellular, Computational and Integrative Biomedicine—CIBIO LaBSSAH, University of Trento, 38122 Trento, Italy; veronica.desanctis@unitn.it (V.D.S.); roberto.bertorelli@unitn.it (R.B.); 9 IRCCS Istituto delle Scienze Neurologiche di Bologna, 40124 Bologna, Italy; melania.giannotta@isnb.it (M.G.); antonella.pini@isnb.it (A.P.); 10 Department of Neurosciences, Biomedicine and Movement Sciences, University of Verona, 37124 Verona, Italy; gaetano.vattemi@univr.it (G.V.); denisealessandra.cassandrini@aovr.veneto.it (D.C.); 11 Immunology Unit, University Hospital, Azienda Ospedaliera Universitaria Integrata, 37126 Verona, Italy; 12 Shoulder & Elbow Surgery Department, IRCCS Istituto Ortopedico Rizzoli, 40136 Bologna, Italy; marco.cavallo@ior.it; 13 IRCCS Istituto Ortopedico Rizzoli, Laboratorio di Immunoreumatologia e Rigenerazione Tissutale, 40136 Bologna, Italy; cristina.manferdini@ior.it (C.M.); gina.lisignoli@ior.it (G.L.); 14 Department of Medical and Surgical Sciences (DIMEC), University of Bologna, 40126 Bologna, Italy; beatrice.fontana10@unibo.it (B.F.); ilaria.pace@unibo.it (I.P.); manuela.ferracin@unibo.it (M.F.); 15 Department of Neurosciences, Rehabilitation, Ophthalmology, Genetics, Maternal and Child Health (DINOGMI), University of Genova, 16132 Genova, Italy; claudio.bruno@unige.it (C.B.); chiara.fiorillo@unige.it (C.F.); 16 Center of Translational and Experimental Myology, IRCCS Istituto Giannina Gaslini, 16147 Genova, Italy; 17 Child Neuropsichiatry Unit, IRCCS Istituto Giannina Gaslini, 16148 Genova, Italy; 18 Institute of Cell Biology, University of Edinburgh, Edinburgh EH9 3BF, UK; e.schirmer@ed.ac.uk

**Keywords:** EDMD, emerin, laminopathies, fibrosis, CRISPR/Cas gene editing, miRNA profiling

## Abstract

Emery–Dreifuss muscular dystrophy (EDMD) is caused by mutations in *EMD, LMNA*, *SYNE1*, *SYNE2*, and other related genes. The disease is characterized by joint contractures, muscle weakening and wasting, and heart conduction defects associated with dilated cardiomyopathy. Previous studies demonstrated the activation of fibrogenic molecules such as TGFbeta 2 and CTGF in preclinical models of EDMD2 and increased secretion of TGFbeta 2 in patient serum. A wide screening of patient cells suggested fibrosis, metabolism, and myogenic signaling as the most affected pathways in various EDMD forms. In this study, we show that alpha-smooth muscle actin-positive myofibroblasts are overrepresented in patient fibroblast cultures carrying *EMD*, *LMNA*, or *SYNE2* mutations, and profibrotic miRNA-21 is upregulated. Upon CRISPR/Cas correction of the mutated *EMD* or *LMNA* sequence in EDMD1 or EDMD2 fibroblasts, respectively, we observe a reduced expression of fibrogenic molecules. However, in patient myoblasts, neither fibrogenic proteins nor miRNA-21 were upregulated; instead, miRNA-21-5p was downregulated along with muscle-specific miRNA-133b and miRNA-206, which have a crucial role in muscle cell homeostasis. These observations suggest that the conversion of laminopathic fibroblasts into a profibrotic phenotype is a determinant of EDMD-associated muscle fibrosis, while miRNA-206-dependent defects of laminopathic myoblasts, including altered regulation of VEGF levels, contribute to muscle cell deterioration. Notably, our study provides a proof-of-principle for the application of gene correction to EDMD1 and EDMD2 and presents EDMD1 isogenic cells that exhibit an almost complete rescue of a disease-specific miRNA signature. These cells can be used as experimental models for studying muscular laminopathies.

## 1. Introduction

Different types of Emery–Dreifuss muscular dystrophy (EDMD) have been identified [1]. EDMD2, the most represented form of EDMD, is caused by *LMNA* gene mutations [2], EDMD1 is linked to *EMD* gene encoding the inner nuclear membrane protein emerin [3], and EDMD4 and EDMD5 are associated with mutations in *SYNE1* or *SYNE2* genes, respectively, encoding nesprin 1 or 2 [4]. Other EDMD forms are linked to *FHL1* and *TMEM43* gene mutations, and similar diseases are due to *SUN1* or *SUN2* gene variants [5,6,7]. EDMD is characterized by Achilles tendons, elbow and neck contractures, and progressive wasting of skeletal muscles associated with cardiac symptoms such as atrial fibrillation, lethal ventricular arrhythmias, and heart failure [1,8]. Symptomatic treatments mitigate orthopedic and cardiac complications, yet a cure is not available for these diseases [1,8]. The pathogenesis of EDMD is not entirely elucidated. In vivo imaging shows fibrotic areas in the myocardium and skeletal muscles at early stages of the disease, while a wide screening of sera from patients affected by *LMNA*-linked muscular laminopathies showed an increase in interleukin 17 and TGFbeta 2 [9]. Moreover, the upregulation of profibrotic molecules including TGFbeta 2, connective tissue growth factor (CTGF), and other triggers of cell fibrotic conversion has been shown in preclinical models [9,10]. On the other end, in EDMD2 myoblasts, dysregulation of mechanosignaling pathways due to altered interplay of the mutated proteins with SUN1 and SUN2 or other components of the LINC platform regulating nuclear mechanobiology has been demonstrated [11,12,13,14].

Many microRNAs have been linked to muscular dystrophies, including those related to nuclear envelope proteins [15,16,17]. A recent study showed that miR-21 contributes to skeletal muscle atrophy and fibrosis in a TGFbeta-dependent manner [18]. In this study, we show an upregulation of the profibrotic miR-21-5p and increased secretion of TGFbeta 2 associated with increased levels of alpha-smooth muscle actin (α-SMA) in EDMD1, EDMD2, and EDMD5 dermal fibroblast cultures. To support the disease specificity of this condition, we produced isogenic cells from EDMD1 mutant fibroblasts, and we permanently knocked down the mutated allele in EDMD2 fibroblasts by the CRISPR/Cas system. In gene-edited EDMD1 and EDMD2 fibroblasts, the physiological condition was rescued, and miR-21-5p levels were restored. On the other hand, different molecular features were observed in EDMD1 and EDMD2 myoblasts, where miR-21-5p was downregulated along with muscle-specific miR-133b and miR-206, which have a crucial role in muscle homeostasis as they contribute to the downregulation of genes that induce muscle atrophy [19]. These results suggest that fibroblasts, but not myoblasts, contribute to tissue fibrosis in the frame of EDMD pathogenesis, while cell intrinsic defects in mechanosignaling [12,13,20], as well as the altered expression of miR-133b and miR-206 [21,22], contribute to muscle fiber deterioration.

## 2. Materials and Methods

### 2.1. Cell Culture

Human fibroblast cultures were obtained from skin biopsies of EDMD patients carrying mutations in *EMD* (EDMD1), *LMNA* (EDMD2), and *SYNE2* (EDMD5) genes, and age-matched healthy donors (control). Human myoblast cultures were obtained from muscle biopsies obtained by the two EDMD1 patients here referred to as EDMD1 #1 and EDMD1 #4. EDMD and control human cell cultures were from the BioLaM biobank approved by the “IOR Ethics Committee” on 5 September 2016. Prot. gen 0018250-01-13. All EU and local ethical rules were respected. A detailed description of each cell culture used in this study is reported in Table S1. The list of samples subjected to gene editing and the mutated sequences is reported in Table 1.

Cells seeded at 10,000/cm^2^ density were grown to confluence in Dulbecco’s modified Eagle’s medium (DMEM), supplemented with 20% fetal bovine serum (FBS) (Gibco Life Technology, Thermo Fisher Scientific, Waltham, MA, USA) and antibiotic/anti-mycotic solution (Sigma-Aldrich, St. Louis, MO, USA). Myoblast cultures were selected based on desmin positivity (at least 70% positive cells). In a subset of myoblast cultures, culture medium was replaced at confluence and cells were allowed to form myotubes for 10 days. Differentiating myoblasts and myotubes were recognized by caveolin 3 positivity. Cells featuring caveolin 3 positivity and at least two nuclei were counted as myotubes.

Confluent fibroblast or myoblast cultures were left in freshly replaced culture medium for 3 days. Then, medium from each sample was collected, centrifuged, and stored at −20 °C until ELISA testing was performed.

Confluent fibroblast cultures were left in freshly replaced culture medium for 3 days and then harvested for miRNome analysis (see below).

HEK293T cells were obtained from the American Type Culture Collection (ATCC #CRL-3216) and were cultured in Dulbecco’s modified Eagle’s medium (DMEM) supplemented with 10% fetal calf serum (FCS), 100 U/mL penicillin, and 100 mg/mL streptomycin (Lonza Ltd., Basel, Switzerland).

### 2.2. Transduction of Fibroblasts and Myoblasts by Lentiviral Vector for EMD Editing

Lenti-hye-A3A-BE4max gRNA to correct the *EMD* mutation carried by EDMD1 #1 was generated from the plasmid lenti-117G-hye-A3A BE4max (#157946; Addgene) digested with PacI and NheI enzymes (NEB, Ipswich, Massachusetts, USA) and self-ligated to remove the 117gRNA. Then, the backbone was linearized with XhoI and the gRNA *EMD* expression cassette was subcloned (Table S2). The resulting lentiviral transfer plasmid was packaged into HEK293T cells by CaPO_4_ transfection protocol, and the produced lentiviral vector (LV CBE) was concentrated by ultracentrifugation [23].

Skin fibroblasts and myoblasts were seeded on 6-well culture plate at a concentration of 1.2 × 10^5^ cells/well and transduced with LV CBE. Untransduced EDMD1 #1 cells were used as a control. The cells were spinoculated for 45 min at 1800 rpm at 20 °C and incubated at 37 °C for six hours. Then, the medium was replaced with fresh medium containing puromycin to select the bulk population transduced by gRNA *EMD* LV. Three weeks after transduction, the cells were harvested and used for genomic and biochemical analysis.

### 2.3. Nucleofection of EDMD1 and EDMD2 Cells

Electroporation of p.L35PinsV *LMNA* mutant fibroblasts and c.650_654dupTGGGC *EMD* myoblasts with Alt-R ribonucleoproteins (RNPs) was performed as described in [24]. Briefly, crRNAs and tracrRNA (IDT Integrated DNA Technologies, Coralville, IA, USA) were resuspended in nuclease-free duplex buffer (IDT Integrated DNA Technologies, Coralville, IA, USA) at a concentration of 100 μM and mixed in equal molar amounts at a concentration of 25 μM, as recommended by the manufacturer. To generate RNPs, 72.5 pmol of annealed crRNA: tracrRNA were mixed with 60 pmol of Alt-R HiFi SpCas9 Nuclease V3 (IDT Integrated DNA Technologies, Coralville, IA, USA) and incubated for 10 min at room temperature. Next, 60 pmol Alt-R Cas9 Electroporation Enhancer (IDT Integrated DNA Technologies, Coralville, IA, USA) was added, as recommended by the manufacturer. Primary fibroblasts or myoblasts were resuspended in 95.5 μL P3 solution from P3 Primary Cell 4D-Nucleofector Kit L (Lonza Ltd., Basel, Switzerland), mixed with 4.5 μL of Alt-R RNP, and electroporated using program CM-138.

### 2.4. Analysis of CRISPR/Cas9 on- and Off-Target Editing

Genomic DNA from primary cells was extracted using the QIAamp DNA micro kit (QIAGEN, Hilden, Germany), according to the manufacturer’s protocol. The genomic regions flanking gRNA target sites were amplified by PCR using Platinum Superfi II DNA polymerase (Thermo Fisher Scientific, Waltham, MA, USA) and the primers used were indicated in Table S2.

Editing efficiency and specificity was assessed by tracking of indels by decomposition (TIDE) analysis on PCR amplicons of the genomic region surrounding the gRNA target site in HD fibroblasts. PCR amplicons of the target regions of the EDMD2 mutant were further cloned in TOPO TA vector [25], Sanger sequenced and analyzed for indels frequency.

For NGS analysis of on-target editing in primary cells, PCR amplicons of the genomic target regions were further amplified by a limited number (n = 8) of PCR cycles to add Illumina Nextera barcodes, using the “2nd amplification” primers listed in Table S2. Libraries were purified using the QIAGEN PCR purification kit (QIAGEN, Hilden, Germany). Equimolar amounts of libraries were mixed, diluted, and sequenced using the Illumina MiSeq system (paired-end sequencing; 2 × 250 bp).

### 2.5. microRNA Profiling from EDMD1 Fibroblast Cell Cultures

#### 2.5.1. RNA Extraction

Total RNA was extracted using the MiRneasy kit Qiagen (Qiagen, Hilden, Germany # 217084) from a total of 5 samples. Specifically, three RNA samples were obtained from EDMD1 fibroblast cultures derived from patients carrying different *EMD* gene mutations (EDMD1 #1; EDMD1 #2; EDMD1 #3), one sample was obtained from EDMD1#1 edited fibroblasts, and one was obtained from control fibroblasts.

#### 2.5.2. Library Preparation and Sequencing

Small RNA-seq was performed using the Qiaseq miRNA Library Kit (Qiagen # 331601) following the manufacturer’s instructions. The quality and concentration of libraries were determined using the High Sensitivity DNA ScreenTape Analysis on the TapeStation 4150 system (Agilent Technologies, Santa Clara, CA, USA). The libraries were diluted to 1.5 pM and sequenced using the NextSeq 500/550 High Output Kit v2.5 75-cycle flow cell (Illumina, San Diego, CA, USA) on the NextSeq 500 platform (Illumina). The sequencing raw data (FASTQ) were analyzed using the QIAseq miRNA Primary Quantification pipeline via the GeneGlobe Data Analysis Center.

Raw counts were normalized using the DESeq2 bioconductor package. MiRNAs with normalized expression > the 40th percentile in at least one sample were selected as expressed. Data analysis was performed using the DESeq2 1.26.0 Bioconductor package within the R version 4.2.1 environment. Differentially expressed miRNAs were identified using a fold change  ≥1.5 and an adjusted *p*-value  <  0.10. The heatmap was generated using the pheatmap package.

#### 2.5.3. microRNA RT-qPCR Analysis

For RT-qPCR analysis of microRNA expression, RNA was extracted from fibroblasts and myoblasts (1-1, 5 × 10^6^ cells) using the TRIzol Reagent (Thermo Fisher Scientific). Confluent cell cultures were used, and triplicate samples from each cell culture were obtained for each analysis. RNA concentration and quality was checked using the NanoDrop 2000c Spectophotometer (Thermo Fisher Scientific). Total RNA was retrotranscribed using the microRNA Reverse Transcription Kit (Thermo Fisher Scientific) and miRNA-specific TaqMan probes (Thermo Fisher Scientific) for miR-21 (ID: 000397), miR-133b (ID: 002247), and miR-206 (ID: 000510). The cDNA (corresponding to 15ng of total RNA) was amplified in duplicate by qRT-PCR on a ViiA7 PCR system using the TaqMan Universal Master Mix and the respective TaqMan assays (Thermo Fisher Scientific). miRNA expression levels were normalized using U6-snRNA and calculated with the formula 2−∆∆Ct.

#### 2.5.4. Cytokine Quantification

Culture media from fibroblast or myoblast samples grown to confluence were used for cytokine assessment. The culture medium was replaced at confluence and the cells were left in the medium for 72 h. Human XL Cytokine 24-plex (#FCSTM18B) and TGFbeta 1, 2, and 3 (#FCSTM17) Luminex Kit Performance Assay kits (Bio-techne) were used to perform cytokine analysis, according to the manufacturer’s guidelines. The magnetic-bead-based antibody detection kits allow for simultaneous quantification of the analytes of interest. The plates were read on the Bio-Plex 200 system (Bio-Rad), powered by Luminex xMAP technology. The concentration of analyte bound to each bead was proportional to the median fluorescence intensity (MFI) of the reporter signal and was determined by the standards provided in the kits (Bio-techne). Data were normalized to the cell number of each sample measured soon after medium collection. Data were expressed as a concentration (pg/mL).

#### 2.5.5. Immunofluorescence Staining

For immunofluorescence analysis (IF), fibroblast and myoblast cultures were fixed with 100% methanol at room temperature for 10 min. After saturation of non-specific binding sites with 4% of bovine serum albumin (BSA) solution for 60 min a RT, coverslips were incubated with primary antibodies overnight at 4 °C overnight or 1 h at room temperature and revealed with FITC- or TRITC-conjugated secondary antibodies diluted 1:200 (incubated for 1 h at RT). The samples were mounted with an anti-fade reagent (Molecular Probes Life Technologies) and observed using a Nikon Eclipse Ni epifluorescence microscope with 40x, 60x and 100x objectives (Nikon, Minato, Tokyo, Japan). The images captured with NIS- Elements 4.3 AR software and were elaborated using Photoshop CS.

#### 2.5.6. Antibodies

The antibodies utilized for immunochemical reactions were anti-emerin, (MONX10804, Monosan, Uden, The Netherlands) used at 1:200 dilution; anti-lamin A/C (E1, Santa Cruz Biotechnology, Dallas, TX, USA) used at 1:500 dilution for IF; anti-caveolin-3 (BD Tranduction Laboratories, NJ, USA) used at 1:200 for IF; anti-desmin (Abcam Ab15200 Cambridge, UK) used at 1:1000 for IF; anti-ED-fibronectin (Sigma-Aldrich, St. Louis, MO, USA) used at 1:100 for IF and 1:1000 for Western blot; and anti-α-SMA (Abcam, Cambridge, UK) used at 1:100 for IF.

#### 2.5.7. Statistical Analysis

For statistical analysis, mean  ±  standard deviation of the values obtained in three independent experiments (n = 3) was calculated. Unless stated differently, statistical analysis was performed by applying Student’s t-test, and statistically significant differences between values are indicated (* *p* < 0.05, ** *p* < 0.01,*** *p* < 0.001 or, **** *p* < 0.0001) ± standard deviation.

## 3. Results

### 3.1. Profibrotic Markers in EDMD

**Figure 1:**
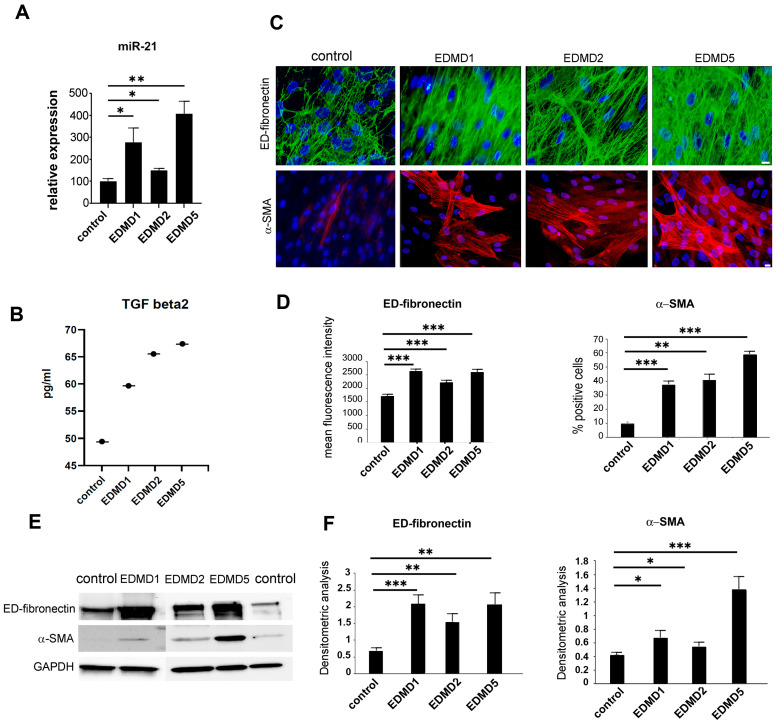
Increase in profibrotic molecules in EDMD fibroblasts. (**A**) qRT-PCR analysis of miR-21 expression; mean values of data obtained in different EDMD1, EDMD2, or EDMD5 patient cultures are reported; (**B**) TGFbeta 2 levels assessed by multiplex immunoassay in medium from healthy donor (control), EDMD1, EDMD2, and EDMD5 fibroblast cultures. (**C**) Immunofluorescence analysis of ED-fibronectin (upper panel) and alpha-smooth muscle actin (α-SMA, lower panel) in control, EDMD1, EDMD2, and EDMD5 fibroblasts. Nuclei are counterstained with DAPI. Representative pictures are shown. Scale bars, 10 mm. (**D**) Quantitative analysis of mean ED-fibronectin and α-SMA fluorescence intensity measured in triplicate samples from different EDMD1, EDMD2, or EDMD5 cell cultures; (**E**) Western blot analysis of ED-fibronectin and α-SMA in control and EDMD1, EDMD2, and EDMD5 fibroblasts; (**F**) Densitometric analysis of ED-fibronectin and α-SMA immunoblotted bands performed in triplicate samples from different EDMD1, EDMD2, and EDMD5 fibroblasts. EDMD1 values in panels A, B, D, and F refer to mean values (three experiments performed in different cell cultures from the same patient) obtained in fibroblasts from patient EDMD1#1 (*EMD* c.1A>G mutation); EDMD2 values in panels A, B, D, and F refer to mean values obtained in fibroblasts from patient EDMD2 (*LMNA* c. 103_104 insCTG mutation); EDMD5 values in panels A, B, D, and F refer to mean values obtained in fibroblasts from patient EDMD5 (*SYNE2* c.2477_2478ins T mutation). Data are reported as means  ±  standard deviation of three independent experiments (n = 3) and statistically significant differences between values calculated by Student’s t-test are indicated by asterisks (*, *p* < 0.05; **, *p* < 0.01; ***, *p* < 0.001).

Our previously published data evidenced a consistent upregulation of TGFbeta 2 in sera from a cohort of EDMD2 patients, while the conditioning of the cells with those sera suggested an underlying profibrotic process as a pathogenetic mechanism [9]. This prompted us to screen the profibrotic miR-21, a TGFbeta regulator [26], and the contractile myofibroblast markers α-SMA and ED-fibronectin in fibroblast cultures obtained from EDMD patients carrying mutations in different genes. As shown in Figure 1A, miR-21 was significantly upregulated in EDMD1, EDMD2, and EDMD5 fibroblasts. Moreover, elevated TGFbeta 2 levels were measured in culture media from laminopathic cell cultures relative to healthy donor cultures (Figure 1B). In EDMD1 fibroblast culture medium, other cytokines, listed in Table 2, were analyzed by multiplex ELISA. Media were added to confluent cell cultures and left for 3 days before collection. We did not find statistically significant differences in the amount of other secreted cytokines, but we did find a trend towards upregulation or downregulation, as shown in Table 2. The behavior (decrease or increase) of each analyzed molecule in EDMD2 patient serum measured in a previous study is also reported in Table 2 for comparison [27]. Of note, only interleukin 6 and TGFbeta 2 levels were elevated both in EDMD1 cell culture medium and EDMD2 patient serum [9,27].

The fibrosis markers, ED-fibronectin and α-SMA, were strongly increased in all examined laminopathic fibroblast cultures, as determined by immunofluorescence (Figure 1C,D) and Western blot analysis (Figure 1E,F). These results indicated the conversion of fibroblasts into contractile myofibroblasts and suggested that profibrotic pathways are activated in the three EDMD forms examined here.

### 3.2. Generation of Isogenic EDMD1 Fibroblast Cultures

To confirm these observations, we generated isogenic control cells by correcting the EDMD1 #1-causing mutations in the *EMD* gene with the CRISPR/Cas system. 

**Figure 2:**
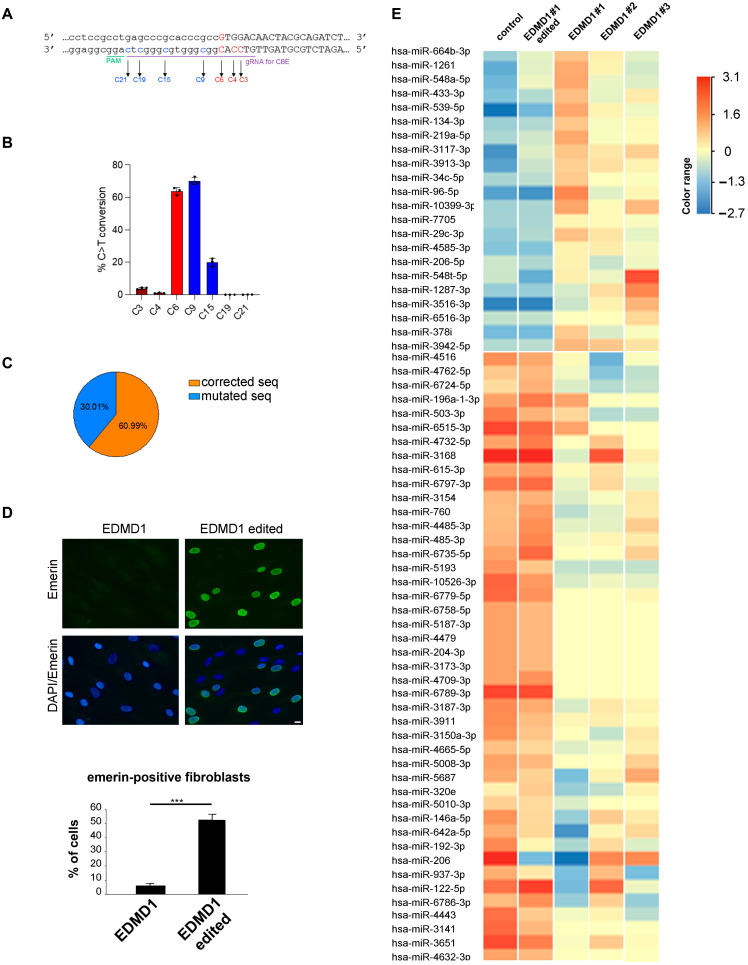
Rescue of emerin expression and miRNome in EDMD1 isogenic fibroblasts by CRISPR/Cas editing of *EMD* gene. (**A**) Representation of gRNA used to target c.1A>G mutation (C6 in red) in EMD gene. C9, C15, C19, and C21 (blue) and C3 and C4 (brown) indicate possible targets of bystander effect of CBE. Capital letters represent part of exon 1 sequence, lowercase indicate 5′UTR region. (**B**) Frequency of on-target (red) and bystander (blue and brown) deamination by NGS analysis of LV CBE-treated patient fibroblasts. (**C**) Percentages of corrected coding sequences over total of genuine reads obtained from patient fibroblasts treated with LV CBE. (**D**) Immunofluorescence analysis of emerin (green) in EDMD1 #1 fibroblasts before (EDMD1) and after gene editing (EDMD1 edited). DAPI (blue) was used to counterstain cell nuclei. Scale bar, 10 μm. Quantitative analysis of emerin mean fluorescence intensity is reported in graph. Data are reported as means  ±  standard deviation of three independent experiments (n = 3) and statistically significant differences between values calculated by Student’s t-test are indicated by asterisks (***, *p* < 0.001). (**E**) Clustering analysis and heatmap representation of differentially expressed miRNAs (fold change > 1.5, adj *p*-value 0.1) in control (control) and isogenic EDMD1 #1 fibroblasts (EDMD1 #1 edited) vs. EDMD1 fibroblasts from different patients (EDMD1 #1, EDMD1 #2, EDMD1 #3). Red color represents expression above average; blue color represents expression below average across all samples.

To specifically correct the *EMD* p.Met1Val gene variant, which is caused by an A to G transition in exon 1 (c.1A>G), the cytidine base editing (CBE) system was exploited in EDMD1 #1 fibroblast cultures (Figure 2A). The gRNA *EMD* (Table S2) designed on the reverse complementary strand carries the mutated C nucleotide at position 6 (C6) and guides the hyperactive A3A-BE4max (hyeA3A-BE4max, [31]) to the 5′-AGG-3′ PAM sequence (Figure 2A). To deliver the CBE system into EDMD1 #1 cells, we packaged hyeA3A-BE4max and gRNA into the single LV CBE. To assess the specificity and efficiency of the CBE, skin fibroblasts from patient 1 were transduced with LV CBE, in triplicate, and analyzed by NGS using the untreated cells as controls. CRISPResso 2.0 analysis on sequence reads scored 63.79% ± 2.34 of C to T transition at the desired position 6, with a negligible level of bystander effect at C3 and C4 (3.98% ± 0.58 and 1.05% ± 0.29, respectively) included in the coding sequence, and relevant, but not risky, bystander effect at C9 and C15 mapping in the 5′UTR sequence of the *EMD* gene (Figure 2B). To better score the frequency of corrected coding sequences, we calculated the reads showing base editing only in the coding sequence out of all base edited reads. Data reported in Figure 2C showed that 60.99% ± 2.40 of the reads contribute to the translation of a functional emerin protein.

In gene-corrected EDMD1 #1 fibroblasts, emerin was detected in more than 50% of nuclei and showed proper nuclear membrane localization (Figure 2D).

To confirm the rescue of wild-type conditions in isogenic EDMD1 #1 fibroblasts, we analyzed the miRNome of three EDMD1 fibroblast cultures derived from patients carrying different *EMD* mutations (EDMD1 #1, #2, #3) as compared to EDMD1#1 isogenic fibroblasts and healthy controls. A miRNA signature emerged from the analysis, as 22 miRNAs were upregulated and 40 miRNAs were downregulated in EDMD1 fibroblast samples from patients compared to the controls (Figure 2E). Importantly, an almost complete rescue of the miRNA expression profile was observed in isogenic fibroblasts (EDMD1#1 edited) (Figure 2E), demonstrating both the efficiency of the gene editing and the impact of emerin restoration on the miRNA expression landscape. Among differentially regulated miRNAs, here we highlight miR-34c-5p, miR-192-3p, and miR-206, which have been involved in muscle homeostasis or regeneration (miR-192-3p); miR-146a-5p, miR-204-3p, and miR-320, which target genes (*FGF2*, *IGFBP2*, and IFITM1, respectively) activating fibrosis or cell proliferation; and miR134-3p and miR-5193, which target AKT and TP53, activating apoptotic and aging processes. A schematic representation of miRNA potentially relevant to EDMD1 pathogenesis and references describing their role in laminopathic conditions, muscle development, or fibrosis is provided in Table 3 (see below).

### 3.3. CRISPR/Cas Editing of EDMD2 Fibroblast Cultures

**Figure 3:**
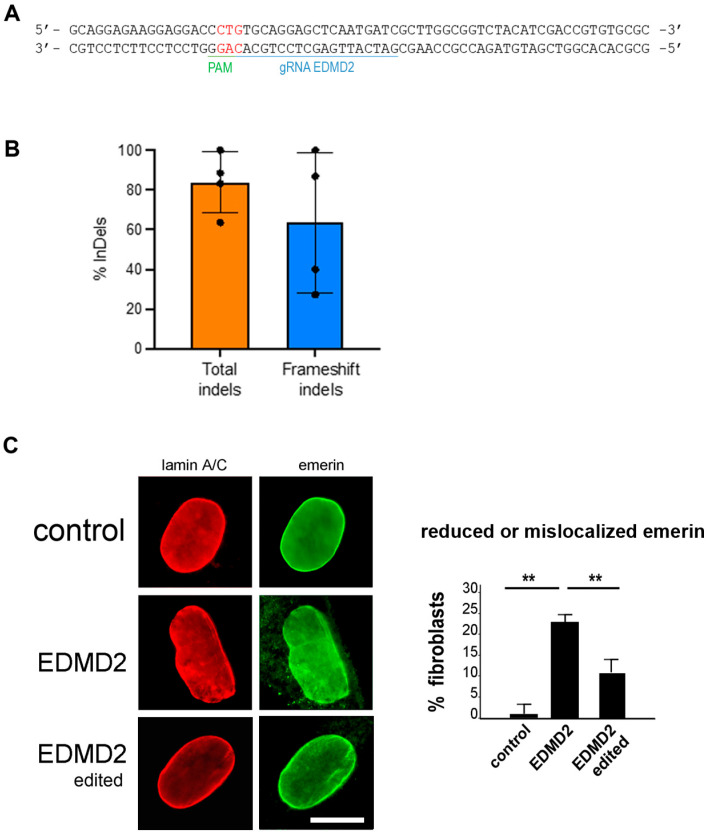
CRISPR/Cas editing of EDMD2 fibroblasts. (**A**) Representation of gRNA used to target c.103_104 insCTG (in red) mutation in exon 1 of *LMNA* gene. (**B**) Frequency of total indels and frameshift editing in EDMD2 fibroblasts treated with RNP carrying Alt-R HiFi SpCas9 complexed to mutation-specific gRNA. (**C**) Immunofluorescence staining of lamin A/C (red) and emerin (green) in control, EDMD2, and gene-edited EDMD2 fibroblasts, showing representative nucleus with reduced nuclear envelope emerin fluorescence/cytoplasmic emerin staining (EDMD2) and rescue upon mutated *LMNA* gene knockdown (EDMD2 edited). Bar, 10 μm. Percentage of cells showing reduced and/or mislocalized emerin is reported in graph. Data are reported as means  ±  standard deviation of three independent experiments (n = 3) and statistically significant differences between values calculated by Student’s t-test are indicated by asterisks (**, *p* < 0.01).

We corrected EDMD2 fibroblasts carrying a dominant heterozygous c.103_104 insCTG (p.L35PinsV) mutation in exon 1 of the *LMNA* gene. To selectively target the p.L35PinsV variant while preserving the wild-type LMNA allele, we designed a mutation-specific gRNA *LMNA* (Table S1) for the SpCas9 nuclease, taking advantage of the PAM 5′-AGG -3′ PAM on the minus strand generated by the insCTG mutation and absent in the WT allele (Figure 3A). We electroporated EDMD2 fibroblasts with ribonucleoparticles (RNPs) carrying the Alt-R HiFi SpCas9 complexed to a mutation-specific gRNA *LMNA* and 48 h later, the editing efficiency was assessed by sequencing. In four independent experiments, the editing efficiency was 83.87%, with the majority of indels leading to a frameshift of the *LMNA* coding sequence, thus resulting in knockdown of the mutant protein (Figure 3B). To confirm editing specificity for the pathogenic variant c.103_104 insCTG (p.L35PinsV), we electroporated healthy donor-derived fibroblasts with RNPs carrying the mutation-specific gRNA *LMNA* and observed the absence of editing, as measured by TIDE analysis, while healthy donor-derived fibroblasts treated with control RNPs including gRNA for the TRAC locus (gRNA TRAC, Table S2) showed approximately 90% editing (Figure S1). EDMD2 fibroblasts showed dysmorphic nuclei and partial emerin mislocalization to the cytoplasm, while only nuclear envelope localization of emerin was observed in gene-edited EDMD2 cell cultures (Figure 3C).

### 3.4. Characterization of Corrected EDMD1 and EDMD2 Fibroblasts

**Figure 4:**
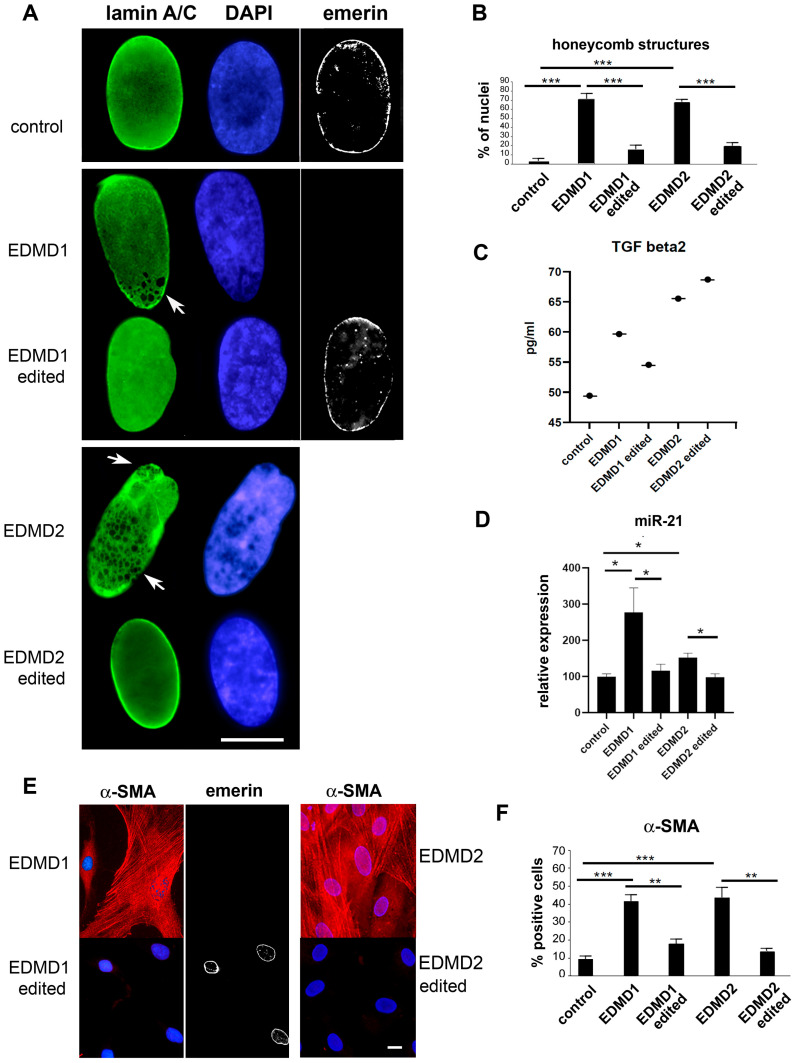
Rescue phenotype in gene-edited EDMD1 and EDMD2 fibroblasts. (**A**) Immunolabeling of lamin A/C (green), DAPI (blue), and emerin (gray scale) in control fibroblasts (control), EDMD1 #1 (EDMD1), isogenic EDMD1 #1 fibroblasts (EDMD1 edited), EDMD2 (EDMD2), and gene-edited EDMD2 fibroblasts (EDMD2 edited). (**B**) Quantitative analysis of nuclear lamina defects (honeycomb structures) in EDMD1 #1, EDMD2, and gene-edited EDMD1 #1 (EDMD1 edited) and EDMD2 fibroblasts (EDMD2 edited) measured as percentage of honeycomb structure-positive nuclei. In total, 200 nuclei per sample were examined. (**C**) TGFbeta 2 levels assessed by multiplex immunoassays in medium from healthy donor (control), EDMD1 #1 (EDMD1), EDMD2, and gene-edited EDMD1 #1 (EDMD1 edited) or EDMD2 fibroblasts (EDMD2 edited). (**D**) qRT-PCR analysis of miR-21 in control, EDMD1, isogenic EDMD1 #1 fibroblasts (EDMD1 edited), EDMD2, and gene-edited EDMD2 fibroblasts (EDMD2 edited). Data are reported as mean ± standard deviation of 2−ΔΔCt values from two independent experiments per group. (**E**) Immunofluorescence analysis of a-SMA (red) in EDMD1, gene-edited EDMD1 #1, EDMD2, and gene-edited EDMD2 fibroblast cultures. Emerin staining of gene-edited EDMD1 #1 nuclei is shown in gray scale. Nuclei are counterstained with DAPI (blue). (**F**) Quantitative analysis of percentage of a-SMA-positive cells in EDMD1, EDMD2, isogenic EDMD1 (EDMD1 edited), and gene-edited EDMD2 (EDMD2 edited) fibroblast cultures. Bars in (**A**,**E**): 10 μm. All experiments are triplicates and data are reported as means  ±  standard deviation of three independent experiments (n = 3), and statistically significant differences between values calculated by Student’s t-test are indicated by asterisks (*, *p* < 0.05; **, *p* < 0.01; ***, *p* < 0.001).

Nuclear dysmorphism or honeycomb structures are typical of laminopathic nuclei and represent a signature of EDMD [32]. The occurrence of honeycomb structures is better observed in cycling cells. Thus, cells seeded 24 h before fixation were used for this analysis. Here, we observed altered nuclear shape and/or honeycomb structures labeled with anti-lamin A/C antibody in 74% of EDMD1 #1 fibroblasts fixed and labeled at 50% confluence (Figure 4A). Notably, these nuclear defects were strongly reduced in isogenic EDMD1 #1 fibroblasts (Figure 4A,B). In EDMD2 fibroblasts carrying the c.103_104 ins CTG *LMNA* mutation, 65% of nuclei showed honeycomb structures, while in cells subjected to gene editing, the percentage of nuclei with honeycomb structures was significantly reduced (Figure 4A,B).

Regarding fibrogenic molecules, TGFbeta 2 levels were reduced in media from isogenic EDMD1 #1, but not in corrected EDMD2 fibroblast cultures (Figure 4C). We further measured TGFbeta 1 and TGFbeta 3 amounts in EDMD2 and edited EDMD2 cell culture media. Neither TGFbeta 1 nor TGFbeta 3 levels were modified in laminopathic samples (Figure S2). Moreover, secreted TGFbeta 1 and TGFbeta 3 amounts were unchanged in gene-corrected cells (Figure S2). However, miR-21 levels were significantly reduced in EDMD1 #1 and EDMD2 fibroblasts subjected to gene correction (Figure 4D) and the percentage of α-SMA-positive isogenic EDMD1 #1 fibroblasts and gene-edited EDMD2 cells was comparable to the controls (Figure 4E,F).

### 3.5. Establishment of Isogenic EDMD1 Myoblasts

**Figure 5:**
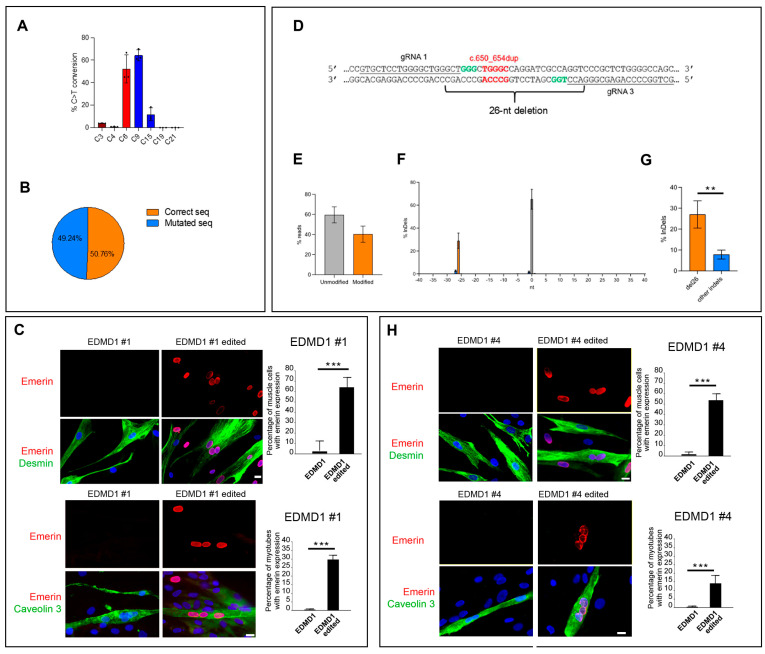
CRISPR/Cas editing of *EMD* restores emerin expression in EDMD1 myoblasts. (**A**) On-target (red) and bystander (blue and brown) deamination in EDMD1 #1 myoblasts treated with LV CBE. (**B**) Percentages of corrected coding sequences (corrected seq) in EDMD1 #1 myoblasts treated with LV CBE (orange) over all edited cells. Percentage of mutated sequences (mutated seq) is also shown. (**C**) Immunofluorescence analysis of emerin (red) and desmin (myoblast marker, green) and emerin and caveolin 3 (myoblast differentiation marker, green) in EDMD1 #1 myoblasts before (EDMD1) or after gene editing (EDMD1 edited). Quantitative analysis is reported in graphs. All myotubes were counted in triplicate samples. (**D**) Representation of gRNAs specific for *EMD* gene carrying five nucleotide duplication (red) in exon 6 of EDMD1 #4. PAM are indicated in green. (**E**) Percentage of unmodified (gray) and modified (orange) sequences of RNP-treated EDMD1 #4 myoblasts. (**F**) Frequency of indels occurring in edited window in EDMD1 #4 myoblasts. Orange bar represents desired 26 nucleotide deletion, and −1 and −27 deletions (blue) indicate unwanted indels caused either by gRNA1 or gRNA3. (**G**) Percentage of sequence with 26-nt deletion (orange) and sequences with other indels (blue) in RNP-treated EDMD1 #4 myoblasts. (**H**) Immunofluorescence analysis of emerin (red) and desmin (myoblast marker, green) and emerin and caveolin 3 (myoblast differentiation marker, green) in EDMD1 #4 myoblasts before (EDMD1) or after gene editing (EDMD1 edited). Quantitative analysis is reported in graphs. All myotubes were counted in triplicate samples. Cell nuclei in C and H were counterstained with DAPI (blue). Scale bars in C and H: 10 μm. Data are means  ±  standard deviation of three independent experiments (n = 3); statistically significant differences between values calculated by Student’s t-test are indicated by asterisks (**, *p* < 0.01; ***, *p* < 0.001).

To correct EDMD1 #1 myoblasts, we availed the CBE system described above (Figure 2A). The CRISPResso 2.0 analysis of NGS reads retrieved from EDMD1 #1 myoblasts treated with CBE showed approximately 52% of the desired C to T conversion, negligible bystander editing in the coding sequence (4% at C3 and 0.8% at C4), and relevant bystander in the 5′UTR that does not affect the full length ORF (Figure 5A), maintained in 50% of the NGS reads (Figure 5B). Immunofluorescence analysis showed emerin-positive nuclei in 65% of EDMD1 #1 myoblasts subjected to gene correction, while more than 25% of myotubes showed emerin staining in nuclei (Figure 5C). To correct the mutation in the *EMD* gene exon 6 of EDMD1 #4 myoblasts, we designed a couple of gRNAs (gRNA1 EMD ex6 and gRNA3 EMD ex6) in opposite orientation to remove 26 nt (del26) of exon 6 and restore the open reading frame (ORF) (Figure 5D, Table S1). EDMD1 #4 myoblasts were co-electroporated with RNPs of the Alt-R HiFi SpCas9 nuclease and the two designed crRNAs, and DNA was used for editing analysis of the target region by NGS. CRISPResso 2.0 analysis of NGS reads indicated that 40.41% ± 8.04 of the reads were edited (Figure 5E); however, deletions of 1 and 27 nt occurred at a detectable level (1.8 and 2.7%, respectively, Figure 5F), indicating that the frequency of reads carrying only the desired del26 editing was 27.01% ± 6.53 over the total EDMD1 #4 sequences scored (Figure 5G). Gene editing restored emerin expression in 52% of desmin positive EDMD1 #4 myoblasts, and emerin positivity was detected in 15% of myotubes (Figure 5H).

### 3.6. Characterization of Isogenic EDMD1 Myoblasts

**Figure 6:**
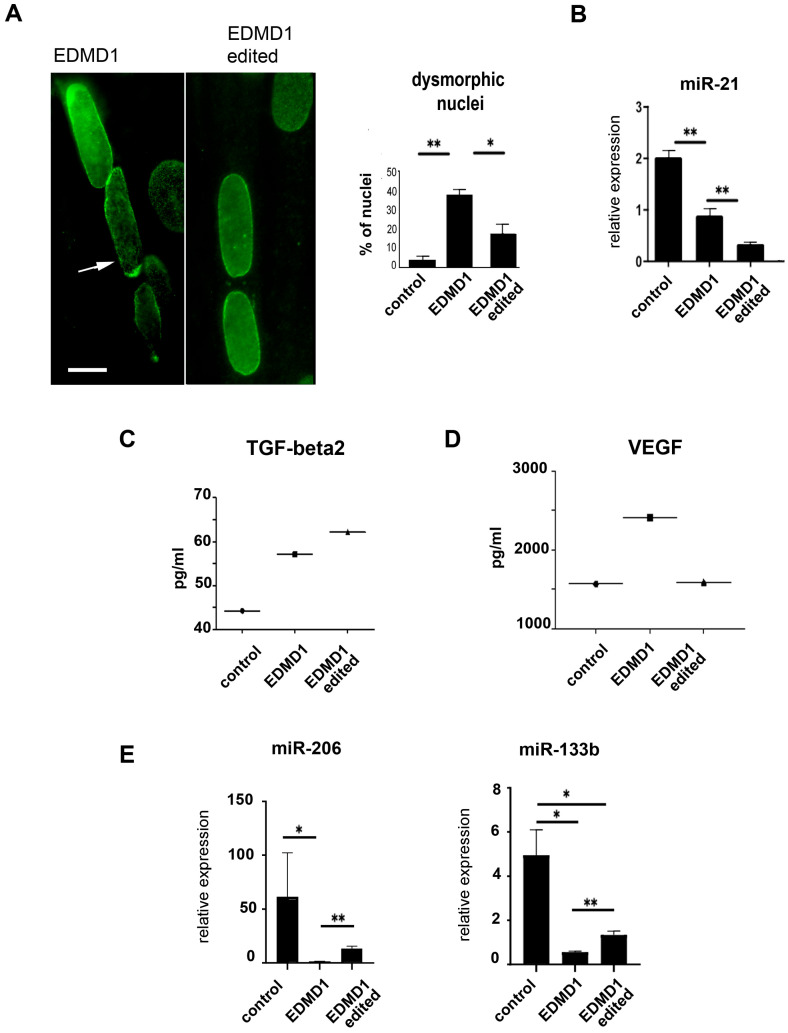
Rescue of cellular phenotype in gene-edited EDMD1 myoblasts. (**A**) Immunofluorescence staining of lamin A/C in EDMD1 #4 (EDMD1) and isogenic EDMD1 #4 myotube nuclei (EDMD1 edited). Percentage of nuclei showing honeycomb structures (arrow) and/or altered nuclear shapes is reported in graph. Bar: 10 μm. (**B**) qRT-PCR analysis of miR-21 expression in control, EDMD1 #1, and isogenic EDMD1 #1 myoblast cultures. (**C**) TGF-beta2 levels measured by ELISA in secretome of control, EDMD1 #1 myotube cultures, and isogenic myotube cultures. (**D**) VEGF levels measured by ELISA in secretome of control, EDMD1 myotube cultures, and isogenic EDMD1 #1 myotube cultures. (**E**) qRT-PCR analysis of myo-MiRs, miR-206, and miR-133b in control, EDMD1 #1, and isogenic EDMD1 #1 myoblast cultures. qRT-PCR data are expressed as means ± standard deviation of 2−ΔΔCt values from two independent experiments per group. All data are means ± standard deviation of three independent analyses performed in different myoblast cultures. Statistically significant differences between values calculated by Student’s t-test are indicated by asterisks (*, *p* < 0.05; ** *p* < 0.01).

To characterize gene-edited EDMD1 myoblasts, we stained lamin A/C in differentiated myoblast cultures and measured the percentage of nuclei showing honeycomb structures or altered nuclear shapes. A significant amelioration of nuclear morphology was assessed in myotubes formed in gene-edited EDMD1 #4 myoblast cultures (Figure 6A).

These data were collected in multinucleated caveolin-positive EDMD1 #4 myotubes. However, due to the higher gene editing efficiency obtained in EDMD1 #1 myoblast cultures, all of the following parameters were measured in EDMD1 #1 samples.

**Table 3:** List of microRNAs potentially relevant in EDMD1 pathogenesis. This table shows differential expression in EDMD1 #1 (up or down); rescue in CRISPR-edited cells; suggested pathway(s); target gene(s) related to proliferation, fibrosis, or myogenesis; references to studies performed in EDMD; and references to studies related to myology or fibrosis.

miRNA	Change in EDMD1 Fibroblasts (F) or Myoblasts (M)	Rescue in CRISPR-Edited EDMD1 Cells	Suggested miRNA Pathway	Target	EDMD Studies	Myology or Fibrosis Studies
miR-21	Up (F, M)	yes	fibrosis	*Smad7* *YAP*	-	[18,33]
miR34c-5p	Up (F)	yes	muscle homeostasis	*nNOS*	-	[34]
miR-133b	Down (M)	yes	muscle homeostasis fibrosis	*CTGF*	[16]	[19,35]
miR134-3p	Up (F)	yes	apoptosis	*AKT*	-	[36]
miR146a-5p	Down (F)	yes	fibrosis	*FGF2*	[15] ***	[37]
miR192-3p	Down (F)	yes	regeneration	*NR3C1* *PIM1*	[17] ****	[38]
miR-204-3p	Down (F)	yes	proliferation autophagy	*IGFBP2*	-	[39]
miR-206	Up/Down (F) * Down (M)	Yes	muscle homeostasis fibrosis	*HDAC4*	-	[19]
miR-320	Down (F)	yes	fibrosis	*IFITM1*	-	[40]
miR-5193	Down (F)	yes	aging	*TP53*	-	[41]

* Different primers (Figure 2E). *** Analysis performed in human myoblasts. **** Analysis performed in human muscle biopsies.

To analyze profibrotic molecules, we measured miR-21 in myoblast culture lysates and TGFbeta 2 levels in the EDMD1 #1 myoblasts medium. Unexpectedly, miR-21 was downregulated in EDMD1 #1 myoblasts and its levels were further decreased upon gene editing (Figure 6B). Moreover, TGFbeta 2 was increased in the EDMD1 #1 myoblast secretome; however, its amount was unchanged upon gene correction (Figure 6C). On the other hand, we observed an increased secretion of vascular endothelial growth factor (VEGF) in EDMD1 #1 myoblast cultures and complete rescue after gene correction (Figure 6D). The latter results showed that the activation of profibrotic pathways does not occur in muscle cells and suggested that cell intrinsic or systemic effects drive pathogenetic pathways in those cells.

As a whole, the miRNome analysis and the qPCR study indicated the role of microRNAs in EDMD1 pathogenetic pathways. Table 3 reports a list of microRNAs here identified in EDMD1 fibroblasts or myoblasts and potentially involved in EDMD pathogenesis due to their role in the regulation of genes and pathways implicated in muscle homeostasis or cell proliferation and profibrotic events.

## 4. Discussion

Previous studies showed increased levels of TGFbeta 2 in EDMD2 patient serum and the activation of profibrotic pathways mediated by TGFbeta 2 and CTGF in preclinical models of the disease [9,10]. Here, we found that a similar pathway could drive the pathogenesis of other EDMD forms. A profibrotic condition was indeed assessed in fibroblasts from EDMD1, EDMD2, and EDMD5 patient biopsies. Interestingly, in all cases, increased TGFbeta 2 secretion was observed in cultured fibroblasts, supporting the view that a TGFbeta-dependent mechanism triggers fibrosis in muscular laminopathies associated with mutations in lamin A/C, emerin, or nesprin 2. Regarding *SYNE 2* mutant fibroblasts, it is worth noting that nesprin 2 silencing has been linked to the inhibition of mechanical stress-related fibroblast trans-differentiation into myofibroblasts [42]. This finding is consistent with our data showing the activation of profibrotic molecules in *SYNE* 2 mutant fibroblasts, directly implying an involvement of mutant nesprin 2 in fibrosis. Recent data show an interaction of nesprin 2 with telethonin at the cardiomyocyte sarcomeres, suggesting that the mutated protein might affect both connective tissue homeostasis and muscle functionality, a hypothesis that warrants investigation in EDMD5 [43].

In the case of EDMD1, the results were supported by comparison between data obtained in patient fibroblasts and their isogenic cells, derived by CBE-mediated correction of the *EMD* gene. Moreover, EDMD2 fibroblasts knocked out in the mutated *LMNA* allele by the CRISPR/Cas system confirmed attenuation of the fibrogenic phenotype. In this study, we found an upregulation of miR-21, which is known to be a target of TGFbeta and trigger profibrotic processes [18]. We hypothesize that the increased α-SMA and ED-fibronectin production we observed in EDMD fibroblast cultures with mutations in different genes may be triggered by either TGFbeta or miR-21 or both depending on the complex interplay of signaling factors determined by diverse mutations. Of note, miR-21 plays an intriguing role in mechanical sensing as a long-term memory keeper of tissue stiffness, linking mechanical conditions to fibrogenic processes [44]. It has been shown that mesenchymal stem cells keep the imprinting determined by stiff substrates (tissues) through miR-21-dependent MRTF-A activation [44]. In the latter condition, α-SMA accumulation is observed, while inhibition of miR-21 impairs the onset of stiffness-dependent fibrotic conversion [44]. Given such complexity and the well-documented involvement of the nuclear envelope in mechano-response, it would not be surprising if different *LMNA*, *EMD*, or *SYNE2* mutations would affect myogenesis and/or muscle functionality through miR-21, leading to a profibrotic environment upon mechanical stress. On the other hand, a loss of miR-21-related mechanosensing could occur in EDMD1 myoblasts, which showed downregulation of this miRNA. Along this line, it has been demonstrated that the mechanosensing transcription factor YAP is aberrantly imported in the nucleus even in the absence of mechanical stimulation in EDMD2 cells [20,45,46].

Along with induced pluripotent stem cells, isogenic fibroblasts and myoblasts represent a good experimental setting for further evaluation of potential biomarkers [47]. Isogenic cells are particularly useful to discriminate disease biomarkers as they avoid bias determined by the different backgrounds of control cells relative to patient-derived cells. In this study, the miRNome screening of EDMD1 fibroblasts and isogenic cells revealed a clear miRNA signature for EDMD1, although a slight variability among patients was also evident. The availability of such cell cultures allowed us to identify miRNA differentially expressed in EDMD1 and an overall signature that is mostly rescued in CRISPR/Cas-corrected fibroblasts. miRNAs involved in the regulation of fibrosis and cell proliferation, including miR-21, miR-146a-3p, miR-206, and miR-320, are particularly interesting in the context of our study. All these miRNAs are downregulated in fibroblasts. However, it must be noted that miR-206 and miR-320 have been shown to act as anti-fibrotic in fibroblasts and pro-fibrotic in other cell types and, most importantly, their role in vivo appears to be tissue-specific [21,40]. Thus, the intriguing possibility of targeting microRNAs for therapeutic purposes must be carefully considered.

Interestingly, in EDMD1 myoblasts, we identified a defective regulation of the miR-206/133b cluster. These miRNAs are selectively expressed in developing skeletal muscle, but not in the heart, and are under the control of myogenic regulatory genes such as MyoD [48]. The downregulation of miR-133b has been shown in a preclinical model of EDMD2 [16]. Of note, miR-133 acts as a repressor of myoblast proliferation and its downregulation could impair cell cycle exit required for myogenic differentiation [49]. In fact, both miR-133 and miR-206 are known to repress histone deacetylase 4 (HDAC4) and induce muscle differentiation, so that their downregulation may impair myogenesis in EDMD1 [21]. In addition, it has been reported that miR-206 modulates VEGF expression in muscle [50] and a recent report shows that muscle stem cells express the VEGF receptors, while VEGF signaling affects myoblast survival [51]. In this context, the downregulation of miR-206 should cause the upregulation of VEGF in EDMD1 myoblasts, thus reducing the viability of muscle precursor cells [51,52]. Regarding other cytokines, we observed a trend for some of them, but the difference in secreted protein levels between wild-type and EDMD fibroblasts or myoblasts did not reach statistical significance (Table 2). However, given the limited number of samples here employed, and based on previously published data showing significantly higher protein levels of TGFbeta 2, interleukin 17, granulocyte colony stimulating factor (G-CSF), interleukin-1 (IL-1), IL-1 receptor antagonist (IL-1ra), and interleukin 4 in serum from a wide cohort of patients affected by *LMNA*-linked muscular laminopathies [27], we suggest that the effect of the muscle environment plays a fundamental role in the pathogenetic mechanisms of EDMD, including the fibrotic process (Table 2). Moreover, the consistent increase in interleukin 6 and TGFbeta 2 in cell culture media and patient serum [9,19] indicates these molecule as candidate biomarkers.

Finally, our results show that fibrogenic molecules including miR-21, ED-fibronectin, and α-SMA are selectively upregulated in fibroblasts, while only TGFbeta 2 was increased in EDMD1 myoblasts. This finding suggests that fibroblasts play a key role in the pathogenesis of EDMD and may represent a target of therapeutic interventions, including those based on gene editing.

## 5. Conclusions

In conclusion, we show that the conversion of fibroblasts into a fibrogenic phenotype is a common feature of EDMD1, EDMD2, and EDMD5 fibroblasts and it involves profibrotic miR-21- and TGFbeta-related events. By focusing on EDMD1 cells, we demonstrated that many miRNAs are affected in EDMD1 fibroblasts, including some miRNAs previously shown to be altered in EDMD myoblasts [15]. Their expression pattern is almost completely rescued upon gene editing, suggesting a direct regulation of the transcriptional landscape by emerin. Interestingly, among myo-miRs, we identified miR-133b and miR-206 as disease targets in EDMD1 muscle precursors and an altered regulatory loop involving TGFbeta 2 in miR-206 downregulation and a downstream effect on VEGF, all of which are events potentially favoring myoblast proliferation rather than differentiation. These data suggest that fibrotic processes occurring in non-muscle cells and altered control of muscle precursor fate favoring proliferation may contribute to EDMD pathogenesis. Our study identifies miR-21 as a differentially regulated miRNA in fibroblasts, potentially linking altered mechanosensing to fibrosis in EDMD. This paves the way to a deeper understanding of EDMD pathogenesis that warrants further investigation. Finally, the correction of the genetic defect in EDMD1 and EDMD2 cells offered a proof-of-principle of future applications of gene editing to treat EDMD. However, the low rate of myoblast differentiation observed in gene-corrected EDMD1 myoblasts suggests that in vivo translation of CRISPR/Cas-based technologies needs further studies.

## Figures and Tables

**Table 1:** List of mutations corrected in this study. Chromosome location, gene, exon number, mutations, disease, and cell type are reported.

Chromosome	Gene	Exon/ Intron	Mutation (Gene)	Mutation (Protein)	Disease	Cell Type
Chr.X	*EMD*	Exon 1	c.1A>G	p.0	EDMD1	Dermal Fibroblasts/ Myoblasts
Chr.X	*EMD*	Exon 6	c.650_654dup	p.Gln219TrpfsX20	EDMD1	Myoblasts
Chr.1	*LMNA*	Exon 1	c.103_104insCTG	p.L35PinsV	EDMD2	Dermal Fibroblasts

**Table 2:** List of cytokines analyzed in this study in EDMD1 #1 fibroblast or myoblast culture media. Cytokine function; trend in cytokine regulation in our experimental conditions; cell type(s) analyzed here; and previously reported trend in cytokine regulation in laminopathic serum are reported.

Cytokine	Function	Trend	Cell type	Changed in EDMD2 Serum [27]
G-CSF	Anti-inflammatory cytokine [27]	unchanged	EDMD1 fibroblasts and myoblasts	Up
IL-6	Pro-inflammatory cytokine, required for myogenesis, drives *LMNA*-dependent senescence pathways [28,29]	Up	EDMD1 fibroblasts	Up
IL-8	Anti-inflammatory and pro-aging cytokine [27]	Down	EDMD1 fibroblasts and myoblasts	Unaffected
IL-9	Pro-inflammatory cytokine	Down	EDMD1 fibroblasts	Up
MCP-1 (CCL2)	Pro-inflammatory cytokine [27]	Up	EDMD1 myoblasts	Unaffected
MIP-1b (CCL4)	Inflammatory chemokine [27]	Up	EDMD1 myoblasts	Unaffected
VEGF	Regulates myoblast survival, is a miR-206 target [21]	Up	EDMD1 myoblasts	Unaffected
TGFbeta 1	Pro-fibrotic factor [30]	Up	EDMD1 fibroblasts	Unaffected
TGFbeta 2	Pro-fibrotic factor—Promotes the alternative activation of macrophages into the M2 subtype, which are anti-inflammatory cells and profibrotic [9,27]	Up	EDMD1 myoblasts and fibroblasts	Up
TGFbeta 3	Involved in adult myogenesis, limits cell fusion [30]	Unaffected	EDMD1 myoblasts and fibroblasts	Down

## Data Availability

The raw data supporting the conclusions of this article will be made available by the authors on request.

## Supplementary Materials

The following supporting information can be downloaded at: https://www.mdpi.com/article/10.3390/cells14171321/s1. Figure S1: CRISPR/Cas editing of HD fibroblasts. TIDE analysis of HD fibroblasts nucleofected with gRNA LMNA or gRNA TRAC as control. Figure S2: TGFbeta levels in EDMD2 and gene-edited EDMD2 fibroblast medium. Table S1: List of cell cultures used in this study. Table S2: List of primers used in this study.
