# Supplementary material for: Profibrotic Molecules Are Reduced in CRISPR-Edited Emery–Dreifuss Muscular Dystrophy Fibroblasts
Source: Cells. 2025 Aug 27;14(17):1321. doi: 10.3390/cells14171321 (PMC12427866; doi:10.3390/cells14171321)
Supplement: Supplementary file 1 [file cells-14-01321-s001.zip › cells-3796678-supplementary.pdf]

Table S1. List of human cell cultures used in this study. All samples belong to the BioLaM biobank. Cell cultures have been established upon mechanical and enzymatic isolation of cells from skin (for dermal fibroblasts) or tibialis muscle biopsies (for myoblasts) obtained from patients or healthy donors of Caucasian ethnicity.

Sample ID in this study	Mutated Gene	Mutated protein	Mutation (nucleotide)	Mutation (protein)	Disease	Patient sex / age	Cell type	Passage number
**EDMD1#1**	*EMD**	emerin	c.1A>G	p.0	EDMD1	Male / 5 years	Dermal fibroblasts	10-15
**EDMD1#1**	*EMD**	emerin	c.1A>G	p.0	EDMD1	Male / 5 years	Myoblasts	2-4
**EDMD1#2**	*EMD* [1, 2]	emerin	c. 740C>T	p.228>stop	EDMD1	Male / 30 years	Dermal fibroblasts	10-15
**EDMD1#3**	*EMD* [3, 4]	emerin	c.59A>G	p.0	EDMD1	Male / 17 years	Dermal fibroblasts	10-15
**EDMD1#4**	*EMD**	emerin	c.650_654dup	p.Glu219TrpfsX20	EDMD1	Male / 3 years	Myoblasts	2-4
**EDMD2**	*LMNA**	lamin A/C	c.103_104insCTG	p.L35PinsV	EDMD2	Male / 3 years	Dermal fibroblasts	10-15
**EDMD5**	*SYNE2**	nesprin 2	c.2477_2478ins T	p.Val827CysfsTer13	EDMD5	Male / 35 years	Dermal fibroblasts	10-15
**control**	-	-	-	-	-	Male / 15 years	Dermal fibroblasts	10-15
**control**	-	-	-	-	-	Male / 9 years	Dermal fibroblasts	10-15
**control**	-	-	-	-	-	Male / 9 years	Dermal fibroblasts	10-15
**control**	-	-	-	-	-	Male / 9 years	Dermal fibroblasts	10-15
**control**	-	-	-	-	-	Male / 9 years	Myoblasts	2-4
**control**	-	-	-	-	-	Male / 13 years	Myoblasts	2-4
**control**	-	-	-	-	-	Male / 35 years	Myoblasts	2-4

*unpublished

References

1. Nigro, V.; Bruni, P.; Ciccodicola, A.; Politano, L.; Nigro, G.; Piluso, G.; Cappa, V.; Covone, A.E.; Romeo, G.; D'Urso, M.; et al. SSCP detection of novel mutations in patients with Emery-Dreifuss muscular dystrophy: definition of a small C-terminal region required for emerin function. *Hum. Mol. Genet.* **1995**, *4*, 2003–2004, https://doi.org/10.1093/hmg/4.10.2003.
2. Maraldi, N.M.; Lattanzi, G.; Capanni, C.; Columbaro, M.; Merlini, L.; Mattioli, E.; Sabatelli, P.; Squarzoni, S.; A Manzoli, F. Nuclear envelope proteins and chromatin arrangement: a pathogenic mechanism for laminopathies.. **2006**, *50*, 1–8.
3. Squarzoni, S.; Sabatelli, P.; Capanni, C.; Petrini, S.; Ognibene, A.; Toniolo, D.; Cobianchi, F.; Zauli, G.; Bassini, A.; Baracca, A.; et al. Emerin presence in platelets. *Acta Neuropathol.* **2000**, *100*, 291–298, https://doi.org/10.1007/s004019900169.
4. Bione, S.; Maestrini, E.; Rivella, S.; Mancini, M.; Regis, S.; Romeo, G.; Toniolo, D. Identification of a novel X-linked gene responsible for Emery-Dreifuss muscular dystrophy. *Nat. Genet.* **1994**, *8*, 323–327, https://doi.org/10.1038/ng1294-323.

Table S2. List of primers used in this study.

Primer ID	Primer sequence (5' to 3')	Application
gRNA_EMD_For	CACCGTCCACGGCGGGTGCGGGCTC	Oligo annealing
gRNA_EMD_Rev	AAACGAGCCCGCACCCGCCGTGGAC	Oligo annealing
gRNA1 EMD ex6	rCrGrUrGrCrUrCrCrUrGrGrGrGrCrUrGrGrGrCrUrGrUrUrUrUrArGrArGrCrUrArUrGrCrU	Nucleofection
gRNA3 EMD ex6	rGrCrUrGrGrCrCrCrCrArGrArGrCrGrGrGrArCrCrGrUrUrUrUrArGrArGrCrUrArUrGrCrU	Nucleofection
gRNA LMNA	rCrGrArUrCrArUrUrGrArGrCrUrCrCrUrGrCrArCGrUrUrUrUrArGrArGrCrUrArUrGrCrU	Nucleofection
gRNA TRAC	rArGrArGrUrCrUrCrUrCrArGrCrUrGrGrUrArCrArGrUrUrUrUrArGrArGrCrUrArUrGrCrU	Nucleofection
EMD_EX1_F	TCGTCGGCAGCGTCAGATGTGTATAAGAGACAGTGCTCGGCCGGTTTTGGTAG	1st PCR amplification
EMD_EX1_R	GTCTCGTGGGCTCGGAGATGTGTATAAGAGACAGCCGCCGCGTACCTACTACAGG	1st PCR amplification
EMD_EX6_F	TCGTCGGCAGCGTCAGATGTGTATAAGAGACAGTCCTACTTCCTCCTCCACCTCTTTTAT	1st PCR amplification
EMD_EX6_R	GTCTCGTGGGCTCGGAGATGTGTATAAGAGACAGCTTCAGCCTGCATGAAGTGGTAAA	1st PCR amplification
N701	CAAGCAGAAGACGGCATACGAGATTCGCCTTAGTCTCGTGGGCTCGG	2nd PCR amplification
N702	CAAGCAGAAGACGGCATACGAGATCTAGTACGGTCTCGTGGGCTCGG	2nd PCR amplification
N703	CAAGCAGAAGACGGCATACGAGATTTCTGCCTGTCTCGTGGGCTCGG	2nd PCR amplification
N704	CAAGCAGAAGACGGCATACGAGATGCTCAGGAGTCTCGTGGGCTCGG	2nd PCR amplification
N705	CAAGCAGAAGACGGCATACGAGATAGGAGTCCGTCTCGTGGGCTCGG	2nd PCR amplification
N706	CAAGCAGAAGACGGCATACGAGATCATGCCTAGTCTCGTGGGCTCGG	2nd PCR amplification
N707	CAAGCAGAAGACGGCATACGAGATGTAGAGAGGTCTCGTGGGCTCGG	2nd PCR amplification
N710	CAAGCAGAAGACGGCATACGAGATCAGCCTCGGTCTCGTGGGCTCGG	2nd PCR amplification
N711	CAAGCAGAAGACGGCATACGAGATTGCCTCTTGTCTCGTGGGCTCGG	2nd PCR amplification
N712	CAAGCAGAAGACGGCATACGAGATTCCTCTACGTCTCGTGGGCTCGG	2nd PCR amplification
S505	AATGATACGGCGACCACCGAGATCTACACGTAAGGAGTCGTCGGCAGCGTC	2nd PCR amplification
S508	AATGATACGGCGACCACCGAGATCTACACCTAAGCCTTCGTCGGCAGCGTC	2nd PCR amplification
LMNA_For	CCTATTAGAGCCTTTGCCCC	TIDE
LMNA_Rev	CCTGATACCCCCACCATTCC	TIDE
TRAC_EX1_For	TTTCAGGTTTCCTTGAGTGGCA	TIDE
TRAC_EX1_Rev	TGGCCATTCCTGAAGCAAGGAA	TIDE

Figure S1

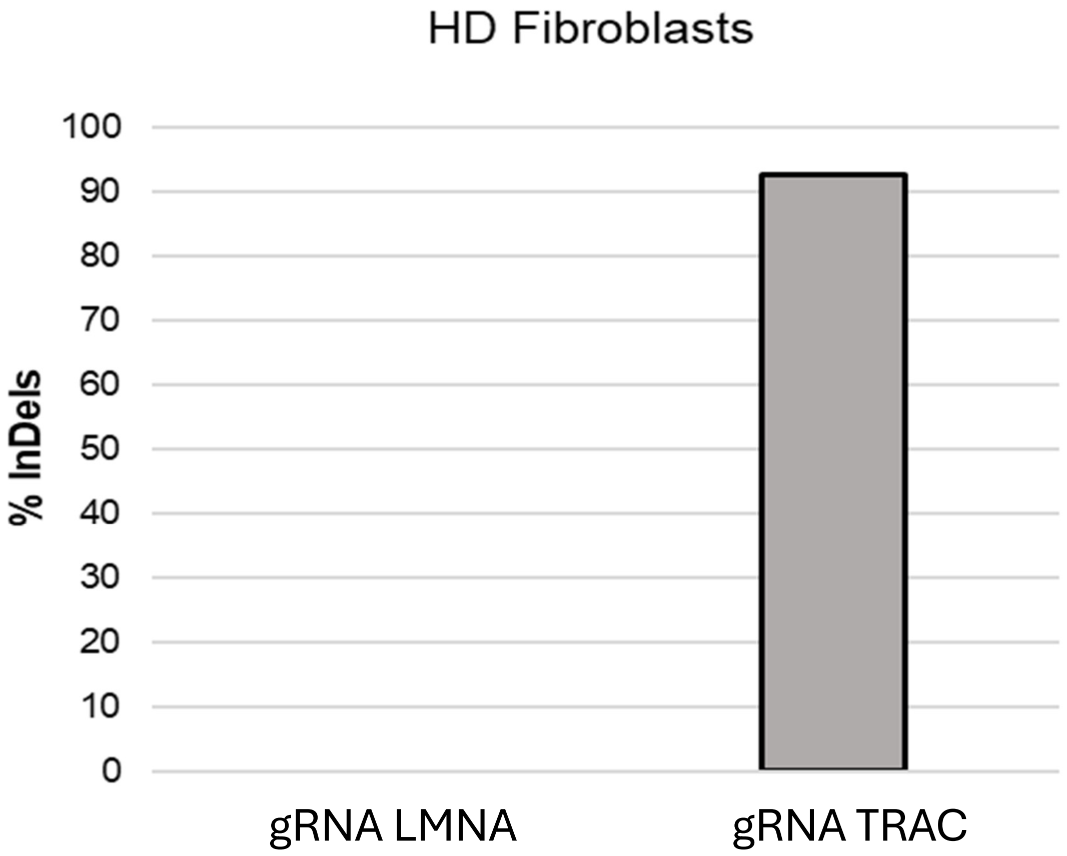

Figure S1. CRISPR/Cas editing of HD fibroblasts. TIDE analysis of HD fibroblasts nucleofected with gRNA LMNA or gRNA TRAC as control.

Figure S2

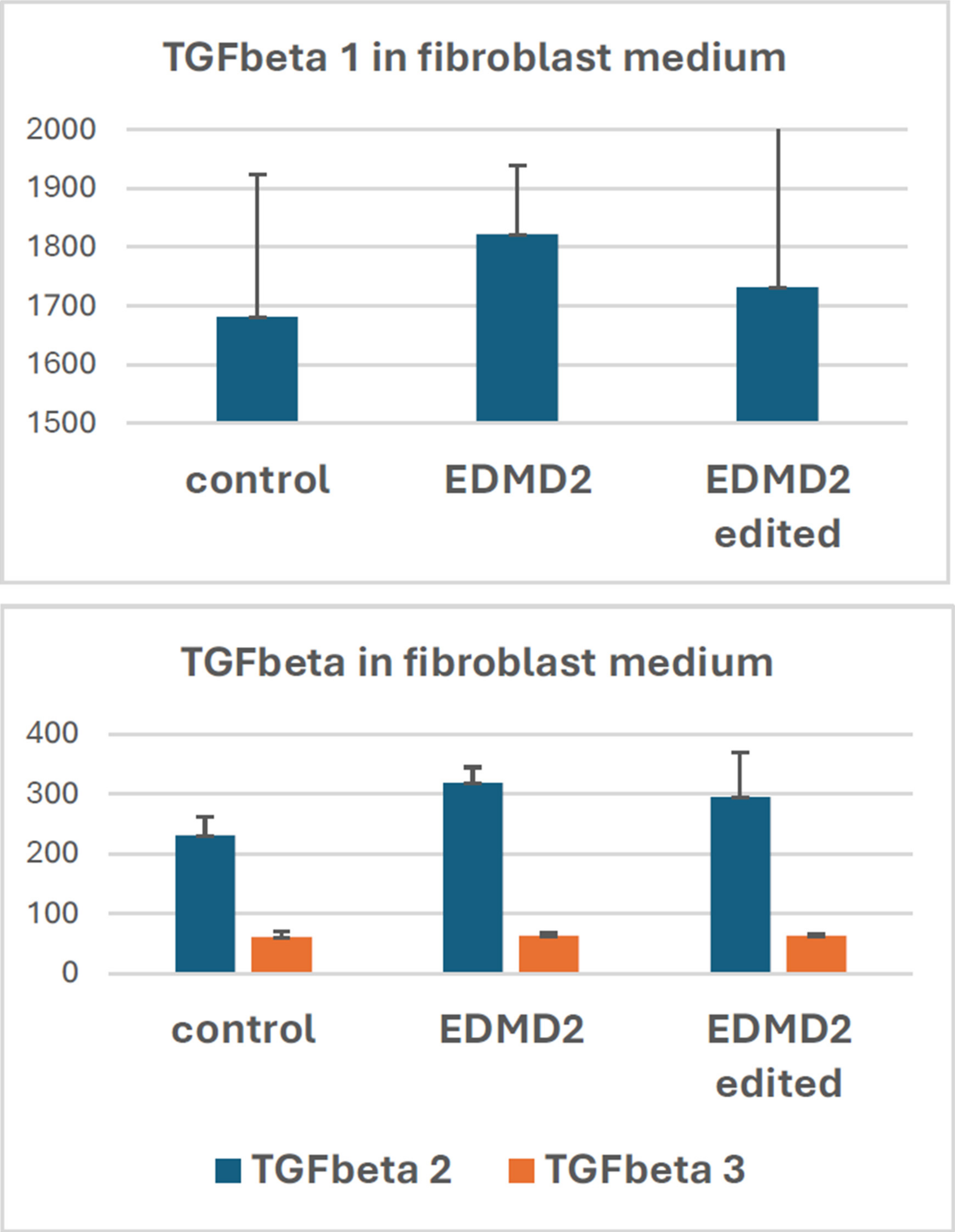

Figure S2. TGFbeta levels in EDMD2 and gene edited EDMD2 fibroblast medium. Data are mean values of triplicate samples +/- standard deviation. TGFbeta 1 and TGFbeta 3 levels in EDMD2 and gene edited EDMD2 were comparable to controls. **The amount of TGFbeta 2 was significantly increased in EDMD2 fibroblast medium, as determined by Student's T-test (*, $p<0.05$).**
